# The Experimental Effects of Acute Exercise on Long-Term Emotional Memory

**DOI:** 10.3390/jcm7120486

**Published:** 2018-11-27

**Authors:** Breanna Wade, Paul D. Loprinzi

**Affiliations:** Exercise & Memory Laboratory, Department of Health, Exercise Science and Recreation Management, The University of Mississippi, Oxford, MS 38677, USA; bcwade@go.olemiss.edu

**Keywords:** amygdala, consolidation, emotional memory, physical activity

## Abstract

Emerging work suggests that acute, moderate-intensity aerobic exercise may help to subserve episodic memory of neutral stimuli. Less investigated, however, is whether acute exercise is associated with enhanced memory recognition of emotional stimuli, which was the purpose of this experiment. A parallel-group randomized controlled experiment was employed. Participants (mean age = 20 yr) were randomized into an exercise (*n* = 17) or control group (*n* = 17). The exercise group engaged in a 15-min bout of moderate-intensity treadmill walking. Emotional memory recognition was assessed via images from the International Affective Picture System, including assessments of varying degrees of valence and arousal. Memory recognition was assessed at 1 day, 7 days, and 14 days post-memory encoding. We observed a significant main effect for time (F(2) = 104.2, *p* < 0.001, η^2^_p_ = 0.77) and a significant main effect for valence–arousal classification (F(4) = 21.39, *p* < 0.001, η^2^_p_ = 0.40), but there was no significant time by group interaction (F(2) = 1.09, *p* = 0.34, η^2^_p_ = 0.03), classification by group interaction (F(4) = 0.12, *p* = 0.97, η^2^_p_ = 0.01), time by classification interaction (F(8) = 1.78, *p* = 0.08, η^2^_p_ = 0.05), or time by classification by group interaction (F(8) = 0.78, *p* = 0.62, η^2^_p_ = 0.02). In conclusion, emotional memory recognition decreased over the 14-day follow-up period and this rate of memory decay was not altered by acute moderate-intensity exercise engagement. We discuss these findings in the context of exercise intensity and the temporal effects of exercise.

## 1. Introduction

Memory function is critical for optimal daily functioning [1,2,3]. Of interest to our research group is whether exercise can subserve memory function. Emerging work from our group [4,5,6,7,8,9,10,11,12] and others [13,14,15,16,17] suggests that acute exercise may help to enhance episodic memory function.

Episodic memory, heavily influenced by the hippocampus, refers to the retrospective recall of past information, typically in a spatiotemporal context. Within the episodic memory domain, emotionally-charged memories, heavily influenced by the amygdala, are often more vividly recalled than non-emotional memories [18]. This may be attributed to various underlying mechanisms, ranging from psychological (increased attention, rehearsal, and elaboration) to physiological attributes. Regarding the latter, key neurotransmitters, including norepinephrine, appear to play an integral role in subserving emotional memories [19,20,21,22,23,24]. Of relevance to the present experiment, acute exercise, which can induce an emotionally-charged response, may also help to facilitate emotional memory function via exercise-induced enhancement in psychological attention [25,26,27,28,29] and modulation of norepinephrine [30].

On these physiological and psychological grounds, it is conceivable that acute exercise may help to subserve emotional memory function. The effects of exercise on emotional memory, however, has not been extensively evaluated [31,32,33], which we have detailed elsewhere [34]. Thus, the purpose of the present experiment was to evaluate the short- and long-term effects of acute exercise on emotional memory.

## 2. Methods

### 2.1. Study Design

A two-arm, parallel-group (between subject) randomized controlled experimental design was employed. This study was approved by the University of Mississippi’s ethics committee (#17-089) and all participants provided written informed consent prior to any participation. Through a computer-generated algorithm, participants were randomized into one of two groups, including an experimental group or a control group. The experimental group walked at a brisk intensity for 15 min, whereas the control group did not engage in any exercise. Participants completed four laboratory assessments. The first assessment involved a training phase where participants viewed 50 images from the International Affective Picture System (IAPS) [35]. Three follow-up assessments occurred for an assessment of long-term emotional recognition memory, including 1-day, 7-day, and 14-day follow-up visits.

### 2.2. Participants

Participants were recruited utilizing a convenience-based sampling approach at the authors’ University. Female participants were exclusively recruited, as sex differences are observed in emotional memory function [36], and exercise-induced changes in cognition may be influenced by sex [37]. Each group included 17 female participants (*n* = 34), which aligns with our previous experimental work on this topic, demonstrating adequate statistical power with this sample size [4,5,7]. Participants included undergraduate or graduate students and were between the ages of 18 and 35 years.

Participants were excluded if they:

- Self-reported as a daily smoker [38,39];
- Self-reported being pregnant [40];
- Exercised within 5 h of testing [15];
- Consumed caffeine within 3 h of testing [41];
- Had a concussion or head trauma within the past 30 days [42];
- Took marijuana or other illegal drugs within the past 30 days [43];
- Had been diagnosed with ADD/ADHD (attention deficit disorder/attention deficit hyperactivity disorder) or a learning disability [44].

### 2.3. Exercise Protocol

Those randomized to the exercise group walked on a treadmill for 15 min at a self-selected “brisk walk”. The minimum speed was set to 3.0 mph. Participants then increased the speed to a pace they perceived as brisk, meaning a pace they would walk as if they were late for catching the bus. This specific exercise protocol has been previously shown to enhance episodic memory function [5]. After the brisk 15-min walk, participants rested in a seated position for approximately 15-min before commencing the initial memory training task (viewing 50 IAPS images). For this 15-min break, participants in both groups (exercise and control) sat prior to the emotional memory assessment of the present study.

### 2.4. Memory Assessments

As stated, participants completed a training phase (baseline assessment) and three follow-up assessments for long-term emotional memory evaluation. For the training phase, participants viewed 50 IAPS images. Among these 50 images, 10 were selected from age- and gender-specific normative data [35] to elicit a negative valence-high arousal state; 10 for negative valence-low arousal; 10 for neutral valence-neutral arousal; 10 for positive valence-high arousal; and 10 for positive valence-low arousal. To confirm that our sample had similar responses to these normative valence-arousal data, which has not always been done in previous studies, our sample rated each of these 50 images during the training phase. Valence and arousal ratings were completed using the Self-Assessment Manikin (SAM) scale. For each image, participants completed the SAM happy/unhappy scale to assess valence (range, 9–1; higher score was a more positive valence) and a SAM excited/calm scale to assess arousal (range, 9–1; higher score was a more excited state).

The training phase (baseline visit) involved viewing the 50 IAPS images, with each image displayed on a computer monitor screen for 6 s. After viewing the image, participants then immediately rated the image via the SAM scale. This process continued until all 50 images were viewed.

After this baseline training visit, participants returned for three testing visits, occurring 1 day, 7 days, and 14 days after the baseline training visit. The procedures for the three follow-up visits were identical. For each follow-up visit, the participant returned to the laboratory and viewed 100 IAPS images. These 100 IAPS images included the 50 images from the training visit as well as 50 new (unseen) images that were matched (to the original 50 images) for valence and arousal levels from the five above-mentioned classifications. This matching occurred by using the age- and gender-specific normative data from the IAPS database [35].

During the three follow-up assessments, participants viewed the 100 images (presented in a random order), with each image displayed on a computer monitor screen for 3 s. After each image, participants selected one of three responses, including “remember”, “know”, or “new”. The “remember” response was defined as “The ability to become consciously aware again of some aspect or aspects of what happened or what was experienced at the time the image was presented. In other words, the image brought back to mind a particular association, image, or something more personal from the time of the study, or something about its appearance or position”. The “know” response was acceptable to select if “You recognize that the image was presented in the initial lab visit, but you cannot consciously recollect anything about its actual occurrence or what happened or what was experienced at the time of its occurrence”. Lastly, the “new” response occurred if “You are certain that you did not previously see the image in the initial lab visit”.

For each of the 3 follow-up assessment periods, five memory recognition metrics were calculated, including the summed recognition score for the images viewed during the training phase, summed recognition score for the images not viewed during the training phase, hit rate score, false rate score, and a discrimination index. For the two summed recognition scores, this involved summing the responses from the recognition memory task, with 1, 2, and 3, respectively, referring to "remember", "know", or "new" (range, 10–30; for each of the 5 valence-arousal classifications). Thus, a higher summed recognition score was a greater indication of the image being perceived as “new”. A higher summed recognition score would be expected for the images not viewed during the training session. A hit rate score was calculated as a rate of correctly indicating that they previously viewed the image during the training session (i.e., that they “remembered” or “knew” they saw the image). A false rate score was calculated as a rate of incorrectly indicating that they previously “remembered” or “knew” seeing an image that was not presented during the training phase. Lastly, the discrimination index was calculated as “hit rate-false rate”.

### 2.5. Additional Measurements

Body mass index was determined at baseline from measured height and weight. Further, heart rate (Polar, F1) was measured throughout the exercise and control protocol. Lastly, habitual engagement in physical activity (min/week) was assessed from the Physical Activity Vital Sign Questionnaire.

### 2.6. Statistical Analyses

All analyses were computed in SPSS (v. 24). A 2 (group) × 5 (valence-arousal classification) × 3 (time) repeated measures (RM) ANOVA was computed. Statistical significance was established as a nominal alpha of 0.05. Partial eta-squared (η^2^_p_) was calculated as a measure of effect size.

## 3. Results

Table 1 displays the characteristics of the study variables. Participants, on average, were 20 years of age, with the entire sample including females. The sample was similarly represented by non-Hispanic whites and non-Hispanic blacks. For both the exercise and control groups, resting heart rate at the baseline visit was 79 bpm; the heart rate in the exercise condition increased to approximately 130 bpm.

As noted previously, we used previously established, normative age- and gender-specific IAPS images to elicit the desired valence–arousal emotional responses. However, to confirm this normative data in our evaluated sample, participants, during the baseline training assessment, rated each image. These data are shown in Table 2; results are shown separately for the exercise and control groups. Generally, results were similar across both groups, with a slight group difference for the negative valence-high arousal classification. However, for the five valence-arousal classifications, the results for our sample aligned with the normative data. That is, the images appropriately altered valence and arousal levels. As an example, for the exercise group, and for the negative valence-high arousal classification, the mean (SD) valence score was 18.0 (6.1) and the mean (SD) arousal score was 58.9 (23.2). For the positive valence-low arousal classification, the mean valence score was 59.8 (14.4), whereas the mean arousal score was 33.9 (15.9). Indeed, a 2 (valence, arousal) × 5 (classifications) ANOVA demonstrated a significant interaction effect (F(4) = 85.8, *p* < 0.001, η^2^_p_ = 0.72).

**Figure 1:**
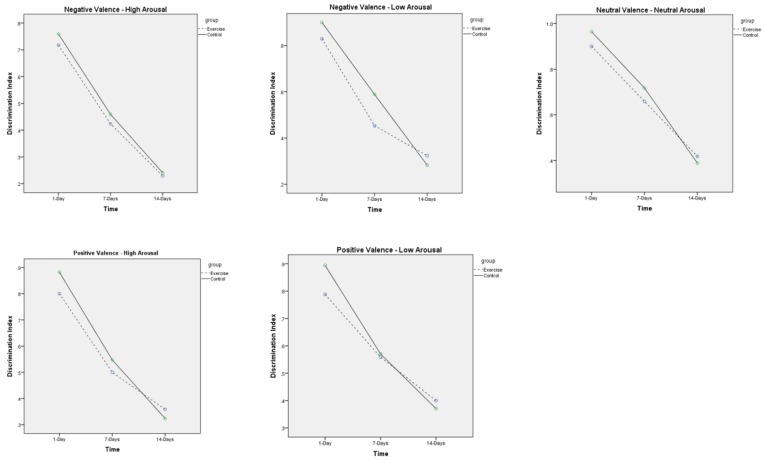
Discrimination index (recognition memory) scores across the three time points (1 day, 7 days, 14 days), for both groups (exercise vs. control), and across all five valence classifications (negative valence-high arousal; negative valence-low arousal; neutral valence-neutral arousal; positive valence-high arousal; positive valence-low arousal). Results show that, across all valence classifications, recognition memory decreased over time, and to the same magnitude for both the exercise and control groups.

The recognition memory scores for both groups (exercise and control), for all five valence-arousal classifications, and across all three follow-up periods (1 day, 7 days, 14 days), are shown in Table 3. Recognition memory scores are displayed for five previously-described metrics. The main analyses for memory recognition was the discrimination index. These results are graphically displayed in Figure 1. As shown in Figure 1, with the specific numeric values displayed in Table 3, for all five valence-arousal classifications, the discrimination index decreased across the three follow-up periods. In general, and for the 1-day, 7-day, and 14-day respective time points, the discrimination index went from an approximate 0.7 to 0.5 to 0.4. Notably, the slope in the declining discrimination index was not different between the exercise and control groups. Regarding our 2 (group) × 5 (valence-arousal classification) × 3 (time) RM-ANOVA, there was a significant main effect for time (F(2) = 104.2, *p* < 0.001, η^2^_p_ = 0.77) and a significant main effect for classification (F(4) = 21.39, *p* < 0.001, η^2^_p_ = 0.40), but there was no significant time by group interaction (F(2) = 1.09, *p* = 0.34, η^2^_p_ = 0.03), classification by group interaction (F(4) = 0.12, *p* = 0.97, η^2^_p_ = 0.01), time by classification interaction (F(8) = 1.78, *p* = 0.08, η^2^_p_ = 0.05), or time by classification by group interaction (F(8) = 0.78, *p* = 0.62, η^2^_p_ = 0.02).

## 4. Discussion

The present study extends previous work that has mostly focused on the effects of exercise on non-emotional episodic memories [6]. Herein, we evaluated whether acute, moderate-intensity aerobic exercise was associated with emotional (and non-emotional) memory recognition. Our results showed that, as expected, emotional memory recognition declined over the 14 day follow-up period. This decline in memory recognition occurred for all valence and arousal stimuli. Additionally, the rate of decline in memory recognition was similar between the exercise and control groups. Thus, we did not observe any beneficial effect of acute, moderate-intensity aerobic exercise on short- or long-term emotional or non-emotional memory recognition.

Albeit limited in investigation, there is some evidence that acute exercise may enhance emotional memory. Using IAPS images, Segal et al. [30] demonstrated that 6 min of high-intensity exercise enhanced short-term emotional memory. Keyan et al. [33] demonstrated that 10 min of higher intensity walking was associated with emotional memory, evaluated up to 48 h post-exercise. Notably, both of these studies implemented the exercise bout after the memory stimuli (i.e., during the early memory consolidation stage). This differs from our study where we implemented the bout of exercise prior to memory encoding. We chose this temporal sequence as our other experimental work on non-emotional episodic memories suggests that acute exercise prior to memory encoding may be optimal when compared to other temporal periods [4,5]. However, the temporal effects of exercise on emotional and non-emotional memory may differ, and, if so, this may explain our null findings. Although speculative, it is conceivable that acute exercise prior to non-emotional episodic memories may be advantageous, as it may help to increase psychological attention and thus enhance the encoding of neutral stimuli. However, this may be less of a concern with emotional stimuli, as the stimuli itself is relatively distinct, which may help facilitate memory encoding. Exercising during the early consolidation period may help to augment levels of key neurotransmitters (e.g., norepinephrine) that are involved in the synaptic consolidation of emotional memories. Further, another possible explanation for our null findings is that we employed a moderate-intensity bout of exercise as opposed to a higher-intensity bout of exercise that was used in previous work on this topic. Although moderate-intensity exercise appears to be optimal for enhancing non-emotional memories [13], it is possible that a higher-intensity bout of exercise during the memory consolidation period is optimal for enhancing emotional memory.

A limitation of this study is the relatively high hit rate across both groups, suggesting that a ceiling effect may have contributed to our null interaction findings between groups. Another limitation of this study includes the relatively small sample size. However, it does align with the sample size of other experiments on this topic [30,31,32,33], particularly between-group experiments with one-time assessments, which is less statistically powerful than our multiple repeat assessment of memory across a two week follow-up period. It is easy to dismiss our null findings by attributing them to the relatively small sample size. However, if this were true, it is likely we would have observed a trend toward significance or a trend toward a point estimate difference between the groups, which was not the case in the present experiment. Our findings point to the suggestion that maybe exercise prior to encoding emotional memories may not enhance emotional memory, and thus, as stated previously, additional work should evaluate the temporal effects of exercise on emotional memory (e.g., exercise occurring during the memory consolidation period). The strengths of this study include the study’s novelty, the comprehensive assessment of emotional and non-emotional memory, the confirmation of the emotional response to the images, and the long-term assessment of emotional memory (up to a 14 day follow-up).

In conclusion, the present experiment does not demonstrate that acute, moderate-intensity walking is associated with enhanced memory recognition of emotional or non-emotional visual stimuli. Additional work on this topic is warranted. Such work should consider evaluating whether there is an intensity-specific effect of exercise on emotional memory, whether exercise temporality influences this paradigm, and whether there is a potential sex-specific effect of exercise on emotional memory.

## Figures and Tables

**Table 1:** Characteristics of the study variables.

Variable	Exercise (*n* = 17)	Control (*n* = 17)
Age, mean years	20.5 (1.2)	20.8 (1.8)
% Female	100.0	100.0
Race/Ethnicity, %		
White	52.9	64.7
Black	47.1	35.3
BMI, mean kg/m^2^	27.6 (6.6)	26.4 (6.2)
MVPA, mean min/week	142.6 (135.6)	162.6 (95.4)
On Birth Control Medication, %	64.7	58.8
Heart Rate, mean bpm		
Resting	79.2 (17.4)	78.7 (16.0)
Midpoint	129.6 (14.1)	83.5 (17.7)
Endpoint	127.2 (18.5)	83.3 (15.3)
2-Min Post	90.6 (19.7)	83.1 (15.3)

BMI, body mass index; MVPA, moderate to vigorous physical activity; values in parentheses are SD (standard deviation) estimates.

**Table 2:** Perceptual assessment of the emotional stimuli during the training session.

Image Ratings During Training Session	Exercise (*n* = 17)	Control (*n* = 17)
Classification		
Negative Valence-High Arousal		
Valence	18.0 (6.1)	24.2 (10.4)
Arousal	58.9 (23.2)	49.5 (20.7)
Negative Valence-Low Arousal		
Valence	27.8 (8.4)	32.0 (12.9)
Arousal	29.4 (15.2)	29.0 (13.5)
Neutral Valence-Neutral Arousal		
Valence	38.7 (8.6)	40.1 (11.0)
Arousal	39.0 (15.7)	33.6 (11.9)
Positive Valence-High Arousal		
Valence	50.2 (6.9)	50.4 (11.2)
Arousal	47.9 (12.7)	43.1 (13.0)
Positive Valence-Low Arousal		
Valence	59.8 (14.4)	60.5 (12.2)
Arousal	33.9 (15.9)	29.3 (12.3)

Point estimates are means and values in parentheses are SD estimates. Valence and arousal estimates range from 9 to 1 for each of the 10 items. The above scores are the summed scores for the 10 items for each classification. Thus, for the above items, the range is 10–90, with higher scores indicative of greater valence/arousal. For valence, 9 is a positive valence (happy, pleased, satisfied content, hopeful) whereas a 1 is a low valence (unhappy, annoyed, unsatisfied, despaired, bored). For arousal, 9 is a high degree of arousal (stimulated, excited, frenzied, jittery, wide-awake, aroused) whereas a 1 is a low degree of arousal (relaxed, calm, sluggish, dull, sleepy, unaroused).

**Table 3:** Recognition emotional memory scores.

Assessment	Exercise (*n =* 17)	Control (*n* = 17)
**1-Day Follow-Up**		
**Negative Valence-High Arousal**		
Summed Recognition Score Viewed Images	13.94 (3.1)	12.65 (2.4)
Summed Recognition Score Non-Viewed Images	27.35 (1.9)	27.47 (2.0)
Hit-Rate	0.94 (0.08)	0.94 (0.06)
False-Rate	0.22 (0.17)	0.18 (0.14)
Discrimination Index	0.71 (0.19)	0.75 (0.13)
**Negative Valence-Low Arousal**		
Summed Recognition Score Viewed Images	16.65 (3.7)	14.41 (3.3)
Summed Recognition Score Non-Viewed Images	28.94 (1.5)	29.18 (1.1)
Hit-Rate	0.92 (0.07)	0.95 (0.06)
False-Rate	0.10 (0.14)	0.06 (0.07)
Discrimination Index	0.83 (0.14)	0.90 (0.09)
**Neutral Valence-Neutral Arousal**		
Summed Recognition Score Viewed Images	14.94 (3.2)	13.41 (3.0)
Summed Recognition Score Non-Viewed Images	29.35 (1.1)	29.88 (0.33)
Hit-Rate	0.95 (0.06)	0.97 (0.05)
False-Rate	0.05 (0.10)	0.01 (0.03)
Discrimination Index	0.90 (0.13)	0.96 (0.06)
**Positive Valence-High Arousal**		
Summed Recognition Score Viewed Images	15.29 (3.6)	12.88 (2.6)
Summed Recognition Score Non-Viewed Images	28.59 (1.5)	29.00 (1.0)
Hit-Rate	0.92 (0.11)	0.97 (0.05)
False-Rate	0.13 (0.14)	0.08 (0.08)
Discrimination Index	0.80 (0.20)	0.88 (0.11)
**Positive Valence-Low Arousal**		
Summed Recognition Score Viewed Images	16.18 (3.7)	15.12 (3.0)
Summed Recognition Score Non-Viewed Images	28.65 (1.8)	29.35 (1.0)
Hit-Rate	0.88 (0.11)	0.95 (0.06)
False-Rate	0.10 (0.11)	0.06 (0.10)
Discrimination Index	0.78 (0.17)	0.89 (0.13)
**7-Day Follow-Up**		
**Negative Valence-High Arousal**		
Summed Recognition Score Viewed Images	14.18 (3.6)	13.06 (3.2)
Summed Recognition Score Non-Viewed Images	21.94 (4.9)	23.24 (6.0)
Hit-Rate	0.94 (0.08)	0.91 (0.08)
False-Rate	0.52 (0.28)	0.45 (0.35)
Discrimination Index	0.42 (0.25)	0.45 (0.30)
**Negative Valence-Low Arousal**		
Summed Recognition Score Viewed Images	15.82 (3.9)	13.82 (3.4)
Summed Recognition Score Non-Viewed Images	23.18 (5.4)	25.12 (4.6)
Hit-Rate	0.92 (0.09)	0.92 (0.111)
False-Rate	0.47 (0.34)	0.34 (0.32)
Discrimination Index	0.45 (0.30)	0.58 (0.28)
**Neutral Valence-Neutral Arousal**		
Summed Recognition Score Viewed Images	15.06 (3.5)	12.47 (2.6)
Summed Recognition Score Non-Viewed Images	25.71 (3.8)	26.47 (3.3)
Hit-Rate	0.97 (0.04)	0.97 (0.04)
False-Rate	0.31 (0.22)	0.25 (0.23)
Discrimination Index	0.65 (0.22)	0.71 (0.22)
**Positive Valence-High Arousal**		
Summed Recognition Score Viewed Images	16.00 (4.0)	12.76 (2.8)
Summed Recognition Score Non-Viewed Images	24.82 (3.9)	24.06 (4.1)
Hit-Rate	0.90 (0.10)	0.97 (0.04)
False-Rate	0.40 (0.28)	0.42 (0.26)
Discrimination Index	0.50 (0.27)	0.54 (0.26)
**Positive Valence-Low Arousal**		
Summed Recognition Score Viewed Images	16.71 (4.5)	13.76 (2.5)
Summed Recognition Score Non-Viewed Images	25.47 (3.7)	25.24 (3.6)
Hit-Rate	0.88 (0.15)	0.93 (0.07)
False-Rate	0.32 (0.22)	0.36 (0.22)
Discrimination Index	0.55 (0.24)	0.57 (0.23)
**14-Day Follow-Up**		
**Negative Valence-High Arousal**		
Summed Recognition Score Viewed Images	15.06 (3.8)	13.59 (4.06)
Summed Recognition Score Non-Viewed Images	19.47 (5.7)	18.76 (7.1)
Hit-Rate	0.89 (0.10)	0.89 (0.16)
False-Rate	0.66 (0.29)	0.65 (0.34)
Discrimination Index	0.22 (0.26)	0.24 (0.28)
**Negative Valence-Low Arousal**		
Summed Recognition Score Viewed Images	15.82 (3.5)	14.41 (4.2)
Summed Recognition Score Non-Viewed Images	21.29 (5.4)	20.29 (6.4)
Hit-Rate	0.93 (0.08)	0.91 (0.14)
False-Rate	0.61 (0.31)	0.62 (0.37)
Discrimination Index	0.32 (0.26)	0.28 (0.30)
**Neutral Valence-Neutral Arousal**		
Summed Recognition Score Viewed Images	15.18 (4.1)	13.35 (3.4)
Summed Recognition Score Non-Viewed Images	22.65 (5.1)	21.65 (6.1)
Hit-Rate	0.94 (0.07)	0.94 (0.07)
False-Rate	0.52 (0.31)	0.55 (0.35)
Discrimination Index	0.41 (0.30)	0.38 (0.30)
**Positive Valence-High Arousal**		
Summed Recognition Score Viewed Images	15.65 (3.9)	13.29 (3.4)
Summed Recognition Score Non-Viewed Images	21.47 (5.2)	20.53 (6.0)
Hit-Rate	0.92 (0.08)	0.94 (0.10)
False-Rate	0.57 (0.30)	0.61 (0.33)
Discrimination Index	0.35 (0.28)	0.32 (0.30)
**Positive Valence-Low Arousal**		
Summed Recognition Score Viewed Images	16.41 (3.9)	14.24 (2.7)
Summed Recognition Score Non-Viewed Images	22.71 (5.8)	22.47 (4.8)
Hit-Rate	0.89 (0.12)	0.92 (0.09)
False-Rate	0.49 (0.34)	0.55 (0.31)
Discrimination Index	0.40 (0.27)	0.37 (0.27)

Point estimates are means and values in parentheses are SD estimates.
